# Machine Learning Algorithms for Adverse Drug Reactions Prediction and Identifying Its Determinants Among HIV Patients on Antiretroviral Therapy in the University of Gondar Comprehensive and Specialized Hospital, in Amhara Region, Ethiopia

**DOI:** 10.1002/hsr2.71306

**Published:** 2025-09-29

**Authors:** Mequanente Dagnaw, Addis Belayneh

**Affiliations:** ^1^ Department of Epidemiology and Biostatistics, Institute of Public Health University of Gondar Gondar Ethiopia; ^2^ Department of Medical Biotechnology, Institute of Biotechnology University of Gondar Gondar Ethiopia; ^3^ Department of Information Technology, College of Informatics, Department of Information Technology University of Gondar Gondar Ethiopia

**Keywords:** adverse drug reactions, Ethiopia, HIV/AIDS, risk‐score

## Abstract

**Background:**

Harmful and unexpected reactions to drugs given at standard dosages using the appropriate administration technique for the goals of therapy, diagnosis, or prevention are known as adverse drug reactions (ADRs). Every medicine has the potential to produce both favorable and unfavorable outcomes. Information regarding the timing of adverse drug reactions and their predictors in adults is not well addressed regarding time and various predictor variables, including the study area, even though three separate studies on the adverse drug reactions of adult patients receiving antiretroviral therapy (ART) have been conducted in Ethiopia.

**Objective:**

To predict adverse drug reactions in HIV patients receiving antiretroviral medication in the University of Gondar Comprehensive and Specialized Hospital using machine learning algorithms.

**Methods:**

Using institution‐based secondary data, patients receiving antiretroviral medication at the University of Gondar Comprehensive and Specialized Hospital between January 11, 2018, and January 10, 2023, were examined. Patient data was extracted from the electronic database using a methodical checklist, and it was then imported into Python version three for pre‐processing and analysis. Then, seven machine learning algorithms for supervised classification were trained to create models. The prediction models were evaluated using F1‐score, AUC, accuracy, sensitivity, specificity, and precision. Association rule mining was used to determine the best rule for the association between independent features and the target feature.

**Result:**

There were 3371 (64.04%) female participants and 1893 (35.06%) male individuals out of 5864 research participants. Among all the chosen classifiers, the random forest classifier (sensitivity = 1.00, precision = 0.987, f1‐score = 0.993, AUC = 0.9989) fared better in predicting ADRs. Based on the importance ranking, the CD4 count was determined to be the most significant predictor feature. The top eight predictors of ADRs were identified by random forest feature importance and association rules as follows: Male, younger age, longer duration on ART, not taking Co‐trimoxazole preventive therapy (CPT), not taking TB (Tuberculosis) preventive therapy (TPT), secondary educational status, TDF‐3TC‐EFV, and low CD4 counts.

**Conclusion:**

Our research shows that HIV patients who are at a high risk of adverse drug reactions and those who can recognize the predictive traits associated with the ADRs can be categorized according to how effectively their ART treatment is working. However, our research may help address the pressing public health issue of diagnosing and treating HIV‐positive individuals.

**Abbreviations**
AIDS: acquired immune deficiency disease syndrome,
ART: antiretroviral therapy
AUC: area under curve
BMI: body mass index
CDC: Center for Disease Control and Prevention
CI: confidence interval
CMV: cytomegalovirus
CNS: central nervous system
CPT: co‐trimoxazole preventive therapy
CRM: cryptococcus meningitis
DHHS: Department of Health and Human Service
EDHS: Ethiopian Demographic and Health Survey
HAART: Highly Active Antiretroviral Treatment
HICs: High‐Income Countries
HIV1, HR: Hazard Ratio, Human Immunodeficiency Virus type 1
IPT: immune peroxidase techniques
KS: Kaposi's sarcoma
LMICS: Low and middle income countries
LMM: linear mixed model
MAC: Mycobacterium Avium complex
MCMC: Markov Chain Monte Carlo
MDGs: Millennium Development Goals
MOH: Ministry of Health
NNRTI: non‐nucleoside reverse transcriptase inhibitor
NRTI: nucleoside reverse transcriptase inhibitor
OIs: opportunistic infections
PCP: Pneumocystis Carinii Pneumonia
PHA: PLHIV, People Living with HIV
PY: person‐year
SNNPR: Southern Nations, Nationalities and Peoples' Region
SSA: sub Sharan Africa
TB: tuberculosis
TPT: TB (tuberculosis) preventive therapy
WHO: World Health Organization.

## 1 Introduction

Human immunodeficiency virus (HIV) infection remains the leading cause of illness and death worldwide [1]. Globally, 37.9 million persons tested positive for HIV in 2018. With an estimated 71% of the world's burden, Sub‐Saharan Africa is the most exposed area. In Ethiopia, 722,248 persons were HIV‐positive in 2017, compared to an estimated 715,404 in 2015 [2].

Anti‐HIV drugs function by preventing the virus from growing, which strengthens the immune system and lowers the chance of infection in anti‐retroviral treatment (ART). It is regrettable that, despite their many advantages, antiretrovirals have been associated with adverse drug reactions, much like many other prescribed drugs. Anti‐HIV‐related ADRs have been found to occur more frequently during the start of ART [3].

The introduction of ART in low‐ and middle‐income nations saved approximately 4.2 million lives [4]. ART‐related side effects, however, can vary from sudden, possibly fatal events to long‐term, covert illnesses; in these cases, antiretroviral treatment must often be stopped immediately, and a new regimen without overlapping toxicities must be started [5].

ADRs are negative and unanticipated reactions to medications administered at recommended dosages using the recommended delivery system for prevention, diagnosis, or treatment. Every drug has the potential to have both beneficial and adverse effects [6]. Assessing the effectiveness and safety of pharmaceutical products on an ongoing basis is critical for patient care in clinical practice. Promoting patient access to safer and more efficient medications will help achieve the long‐term goal of continuous assessment of pharmaceutical benefits and risks [7].

The overall incidence rate of ADRs in Malawi was 9.5% [8]. Thirty percent of people living with HIV (PLWHAV) in India experience ADRs in the first 6 months of treatment, and about 70 percent have them in the next 6–12 months [9]. Because ADRs have the potential to seriously injure patients, there is a need to increase knowledge of how they impact patient care and public health. Several local conditions, including the high prevalence of HIV/AIDS, tuberculosis, poor health care, and patient illiteracy, are responsible for Ethiopia's high rates of drug‐related morbidity and mortality [10].

ART patients saw an incidence of adverse drug reactions ranging from 4.3% to 90%, as reported by multiple international research studies [11]. The onset of ADR has been associated with several factors, including age, gender, ART regimen, length of treatment, prophylaxis against opportunistic infections, WHO clinical stage, disease biomarkers, and body mass index [11]. ADRs may be impacted by the intricacy of anti‐HIV drugs, as well as compromised immune systems brought on by infections [12].

Medical decision‐making can make use of patient‐level data‐driven clinical prediction models. Classical statistical modeling was designed for data with a few dozen input variables and sample sizes that would be considered moderate to medium. This intricacy of data may make traditional statistical reasoning more challenging to handle. As a result, machine learning was used with greater success [13]. Large datasets can be analyzed using machine learning techniques to identify trends, predict the effectiveness of HIV therapy in the future, and more. By evaluating vast amounts of data and enhancing prediction skills, machine learning predictive algorithms can enhance the standard of treatment and anticipate the needs of HIV patients [14]. Furthermore, HIV patients who are at a high risk of stopping their treatment and experiencing adverse drug reactions (ADRs) can be identified using machine learning can quickly and accurately absorb information from subject matter experts, then apply that knowledge to identify HIV risk behaviors across a large data set [15].

Ethiopia is experiencing a severe HIV pandemic and an increase in ADR [16]. Despite these facts, researchers have only used classical statistical methods to determine the causes of ADRs using data from this country [17]. Even while previous studies looked at the causes of ADRs in various regions of Ethiopia, many of them employed classical statistical models to find significant predictors of ADRs. Despite many previous studies identifying risk factors for ADR in adult HIV patients, the characteristics affecting the ADR syndrome still need to be clarified. Our study, which models “comorbidities and laboratory tests” together, provides low‐cost, rapid, and reliable results on ADR syndrome in HIV patients. Therefore, the purpose of this study was to assess Machine Learning Algorithms for Adverse Drug Reactions Prediction and identify its determinants among HIV patients on antiretroviral therapy (ART) in the University of Gondar Comprehensive and Specialized Hospital, in Amhara Region.

## 2 Methods

### 2.1 Study Setting

This study was conducted in in the University of Gondar Comprehensive and Specialized Hospital, in Amhara Region using institution‐based secondary data, patients receiving antiretroviral medication at the University of Gondar Comprehensive and Specialized Hospital Between January, 2018, and January, 2023, were examined.

### 2.2 Data Source

This study used institution‐based secondary data, patients receiving antiretroviral medication at the University of Gondar Comprehensive and Specialized Hospital between January, 2018, and January, 2024, were examined and included in this study. Patient data was extracted from the electronic database using a methodical checklist, and it was then imported into Python version three for pre‐processing and analysis.

### 2.3 Sample Size and Sampling Procedure

The model development and prediction of ADRs included 5864 adult HIV‐positive patients from the ART clinic at the University of Gondar Comprehensive Specialized Hospital. All ART patients receiving treatment at the time of the study were considered.

### 2.4 Inclusion and Exclusion Criteria

All adult patients who were HIV‐positive and had undergone at least 6 months of ART, received viral load testing, and had their treatment records stored in the computerized database at the University of Gondar Comprehensive and Specialized Hospital were included. However, records with substantial missing characteristic data, including CD4 counts, Adherence, and WHO Stage, were excluded.

### 2.5 Data Collection Tools and Procedures

The University of Gondar Comprehensive and Specialized Hospital's ART clinic served as the primary data source for this study. It had previously maintained records on an electronic database with details on patients who tested positive for HIV. Its distinct ART and medical registration numbers (MRN) aid in its identification. A standardized checklist prepared in English was used to collect data from the computerized database for 5264 adult HIV‐positive persons out of all ART patients. It was modified from the admission and follow‐up form for the ART clinic provided by the Ethiopian Federal Ministry of Health [18]. The electronic database contained multiple tables with various feature integrations. After consulting with subject‐matter experts, the features were discovered and classified into four groups: treatment‐related, hematologic and immunological, clinical, and sociodemographic. During data extractions, characteristics that contained personally identifying information, like patient names and phone numbers, were eliminated. Twenty features and one goal feature were obtained from this investigation once all stages of data gathering were finished.

### 2.6 Method of Building a Predictive Model

The procedure for creating a model or mathematical tool that produces a precise prediction [19]. This is the definition of predictive modeling. Classification algorithms are supervised learning techniques that divide a batch of data into multiple groups. In this study, seven supervised classification methods were applied. Assistive vector machine, random forest, decision tree, logistic regression, gradient boosting [20], K‐nearest neighbors [21]. And XG‐Boost was a few of the machine learning techniques used for categorization. During the literature review, it was discovered that these algorithms work better for classification problems in the healthcare area. The algorithms were chosen based on their accuracy, training duration, ability to handle missing data, and ease of interpretation and learning.

### 2.7 Performance Evaluation for Predictive Model

The performances of each model are assessed and contrasted after training. Based on the confusion matrix, the performance of the prediction models was assessed. To evaluate the model's performance, this study used precision, sensitivity, specificity, F1‐score, and the area under the receiver‐operating characteristic (AUC‐ROC). The ability of a binary classifier to predict classification outcomes is assessed using the well‐liked and potent performance indicator known as AUC‐ROC. Between 0.5 and 1.0, the ability to anticipate was either nonexistent or exceptionally good. The confusion matrix is a common performance measuring tool used in machine learning classification tasks and is used to express a model's output as a binary class. This study's determination of the sensitivity, precision, F1‐score, and performance measures of accuracy depended heavily on the confusion matrix. The predictions that make up the matrix have been tallied into a total number of right and wrong predictions.

### 2.8 Data Quality Assurance

An essential first step in evaluating the quality of the data is data quality assurance. The primary investigator trained the ART data clerk for a full day during the initial round of data collecting from the computerized database. To guarantee that the feature range values were accurate and consistent throughout the entire set of data, he saw each step of the identified record extraction procedure. To ensure consistency and similarity, the lead investigator randomly cross‐checked the patient records and verified the data's accuracy regularly.

### 2.9 Data Management and Analysis

To make pre‐processing the data set in Python version 3 easier, the patient data were extracted from the electronic database in Microsoft Excel format and converted to comma‐separated values (CSV). To create predictions, machine learning requires a high‐quality data set. Consequently, managing the missing data is one of the most important pre‐processing procedures for the data set. The data set's missing values were handled via imputation. Thus, basic imputation techniques were used in this study to deal with the missing values. The data set's missing values were imputed using the Simple Imputer class from the scikit‐learn module [22]. Data encoding is a critical step in the pre‐processing stage of data. Categorical variables were encoded using both label and one‐hot encoding techniques. If a value is discrete rather than continuous and falls into one or more categories, it is said to be categorical. In this experiment, categorical variables were encoded using a single‐hot encoding approach. In a single hot encoding, a number between 0 and 1 is used in place of the category values.

## 3 Result

### 3.1 Baseline Socio‐Demographic and Clinical Characteristics of HIV/AIDS Patients on Art Treatment

The final study included a total of 5864 HIV‐infected adults receiving ART 2990 people, or 51% of the study participants, were female. 3401 (62%) of the participants were between the ages of 15 and 26 at the time they were enrolled in ART therapy. Most, 286 (42% of the overall sample) of the study participants were orthodox. Regarding their level of education, 282 (41%) of the patients had only finished high school (Table 1).

**Table 1:** Socio‐demographic characteristics/variables of HIV/AIDS patients on ART treatment at University of Gondar Comprehensive Specialized Hospital (*n* = 5864).

Variables	Category	Frequency(N)	Percentage (%)
Sex	Female	2990	51
	Male	2874	49
Age	15–25	3401	62
	26–45	2463	38
Religion	Orthodox	2463	42
	Muslim	1817	31
	Protestant	1584	28
Education	No education	1348	23
	Secondary	2111	36
	Diploma and above	2405	41
Occupation	Government	4515	77
	Self employed	1349	23
Residence	Urban	4808	82
	Rural	1056	18
House hold	1–2	997	17
	3–5	3753	64
	6–8	1115	19
Care giver	Yes	4573	78
	No	1291	22
Disclosure	Disclosed	5277	90
	Not disclosed	587	10
Parent HIV status	Positive	3166	54
	Unknown	2698	46

### 3.2 Data Pre‐Processing Results

**Figure 1:**
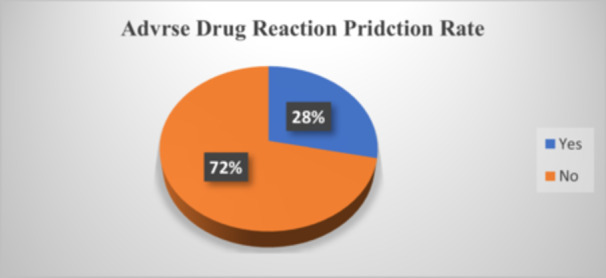
The distribution of the ADR prediction proportion of the Machine learning algorithm.

A classification data set with skewed class proportions is called imbalanced. Classes that make up a large proportion of the data set are called majority classes. Those that make up a smaller proportion are minority classes. Data imbalance usually reflects an unequal distribution of classes within a data set. So, in our study, there is an imbalance data, most of the data are not opportunistic infections, i.e., 0 and a very few classes are opportunistic infections i.e., 1 as shown (Figure 1).

And also, the distribution between some independent with dependent variables was as shown in the diagram.

### 3.3 Model Building and Model Evaluation

**Figure 2:**
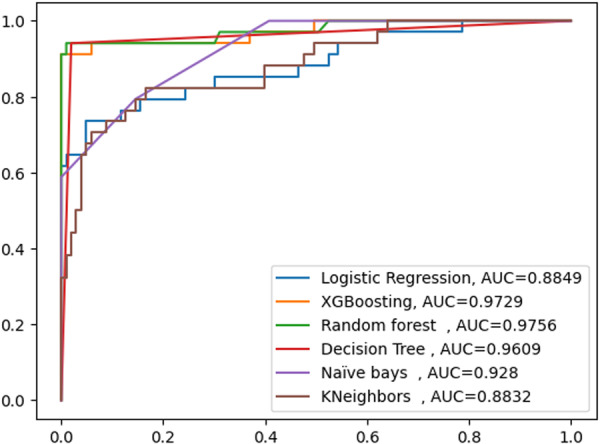
AUC curve for ADRs prediction performance model using unbalanced data set.

This study carried out several trials to build a model that might predict whether opportunistic infections or not in patients receiving ART. We conducted two experiments. these were the training of several classification algorithms using an unbalanced data set, and also the second experiment were identification of the best model using a balanced sampling technique. The imbalanced data set with default hyperparameter tuning performed worse than the balanced data set from all classifiers. Look at Figure 2, AUC for ADRs Prediction performance model using an unbalanced data set (Figure 2).

Therefore, according to the AUC curve, the value of AUC was the same in all classifiers except K‐Neighbors. The classifier was a Random Forest classifier, XG‐Boosting classifier, Logistic Regression classifier, K‐Neighbors classifier, Naïve bays and decision Tree classifier. Thus, the value AUC Random Forest classifier, XG‐Boosting classifier, Logistic Regression classifier, Naïve bays classifier, Naïve bays and Decision Tree classifier were almost 100% but the value AUC K‐Neighbors was 88.32%. In this case accuracy, precision, recall, f1‐score, and AUC were the same various machine learning classification methods to the unbalanced data.

By comparing the model's performance using the balanced data set, the Random Forest classifier outperforms other methods in terms of accuracy and significance of results. While the tests were running, all hyper‐parameters for each approach were kept at their default settings. In this study, the models' performance was evaluated using the AUC curve accuracy, precision, Recall, and f1‐score metrics. The AUC curve in Figure 2 demonstrates that the random forest classifier (AUC = 0.9756) outperformed all other classifiers, with the comparisons of XG‐Boost classifier (AUC = 0.9729), Decision Tree (AUC = 0.9609), Naïve Bays (AUC = 0.928), Logistic Regression (AUC = 0.8849), and K‐nearest Neighbor (AUC = 0.8832). Table 2 performance evaluation findings for the balanced data set demonstrate that each model's classifier produced unique outcomes. The accuracy, precession, recall and f1‐score of the random forest classifier were 97.63%, 97.46%, 99.22% and 98.33% respectively. With a recall of 99.22%, accuracy of 97.45%, and f1‐score of 98.21% the XG‐Boost classifier performed well. Accuracy, precession, recall and f1‐score for the decision tree classifier were 95.99%, 96.44%, 97.93% and 97.18%, respectively. Generally speaking, the classifiers are thought to have shown a respectable performance (Table 2).

**Table 2:** Performance evaluation all predictive models.

	Predictive mode											
Evaluation matrix	Logistic regression		Decision tree		Random forest		XG‐boost		Naïve bays		K‐Neighbors	
	Predicted		Predicted		Predicted		Predicted		Predicted		Predicted	
	No	yes	No	yes	No	yes	No	Yes	No	yes	No	Yes
Metrics												
Accuracy	94%	94%	97%	97%	95%	95%	96%	96%	81%	81%	83%	83
Recall	93%	95%	95%	99%	91%	99%	93%	97%	74%	87%	66%	97%
Precision	94%	94%	98%	96%	98%	93%	96%	94%	82%	80%	95%	78%
F‐score	94%	95%	97%	97%	95%	96%	96%	97%	78%	83%	78%	87%
AUC‐score	0.8849	0.9805	0.9959	0.9959	0.9349	0.9349	0.992	0.992	0.9572	0.9572	0.8905	0.8905

### 3.4 Random Forest Model Performance

**Figure 3:**
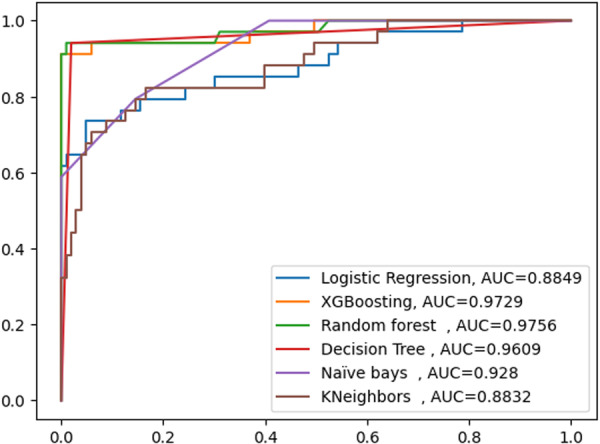
AUC curve for ADRs Prediction performance model using unbalanced data set.

The purpose of this experiment was to assess how effectively various classifiers predicted ADRs. This analysis's goal is to assess how accurately the chosen classifier's predictions were made. In comparison to other chosen classifiers, the random forest classifier performed fairly well on the balanced data set. The best model was selected, and then hyper‐parameter tuning and feature selection were done. Chi‐Squared test and Pearson correlation methods were used for feature selection. Comparing the model's performance required establishing a key prediction of ADRs (Figure 3).

## 4 Discussion

To reach the 95‐95‐95 targets at each level of treatment, Ethiopia is increasing up efforts to identify patients who require more services and treatments [23]. The purpose of this work was to develop the most effective supervised machine learning classifier and to forecast and identify the predictors of ADRs. With the aid of electronic demographic, clinical, and treatment data, seven supervised machine learning classification algorithms logistic regression, K‐Nearest neighbors' classifier, decision tree classifier, random forest classifier, gradient boosting classifier, XG‐Boost classifier, and assistive vector machine were used to forecast ADRs in patients receiving ART [24].

The aforementioned models were picked to const AUC and verify the optimal predictive model employing the key predictors, which was increase model prediction accuracy and generalizability [25]. Split stratified 12‐fold cross‐validation with default hyperparameter tuning was used to train the classifiers on a batch of training samples. To determine the optimal accuracy, numerous tests were conducted using both balanced and unbalanced datasets. Unbalanced data produced low‐performance indicators. Thus, this study compared several methods for addressing unbalanced data. The results of our study show that it is possible to identify HIV patients who are at a high risk of ADRs and to identify the predictive characteristics connected to ADRs using machine learning approaches. Although additional improvements are required, machine learning methods appear to be useful for risk prediction and categorizing ART treatment failure [26]. However, the critical public health issue of locating and treating HIV‐infected patients may be helped by our study methodology. Among chosen classifiers random forest classifier was best. This finding is similar with the previous study [27]. The reason is random forest classifier highly reliable and accurate for a wide variety of predictive modeling tasks.

Clinicians who provide care for HIV patients can benefit greatly from machine learning. The suggested approach has the best AUC, accuracy, precision, sensitivity, and specificity for predicting ADRs in HIV patients. This forecast aids in maximizing the use of hospital resources to treat high‐risk patients, deliver better care, and lower the number of medical errors in ART clinics brought on by exhaustion and lengthy workdays. The quality of care and patient survival may both be enhanced by using efficient predictive models [28]. Therefore, identifying HIV patients at high risk of ADRs and implementing the most efficient supportive and therapeutic regimens can benefit greatly from our investigation of prediction models of ADRs. By offering quantitative, objective, and evidence‐based models for risk classification, prediction, and eventually care planning, this could lessen uncertainty. Additionally, the results of this study may give medical professionals improved methods for minimizing problems and enhancing HIV patients' chances of surviving.

## 5 Conclusion

Our research shows that HIV patients who are at a high risk of adverse drug reactions (ADRs) and those who can recognize the predictive traits associated with the ADRs can be categorized according to how effectively their ART treatment is working. However, our research may help address the pressing public health issue of diagnosing and treating HIV‐positive individuals. Random forest classifier was best classifiers among the chosen one.

## Author Contributions

**Mequanente Dagnaw:** conceptualization, methodology, software, formal analysis, visualization, data curation, supervision. **Addis Belayneh:** investigation, validation, visualization.

## Disclosure

The lead author Mequanente Dagnaw affirms that this manuscript is an honest, accurate, and transparent account of the study being reported; that no important aspects of the study have been omitted; and that any discrepancies from the study as planned (and, if relevant, registered) have been explained.

## Ethics Statement

The Public Health Institute, the College of Medicine, and Health Sciences, University of Gondar's ethical review committee granted clearance and approval to conduct the research under the reference letter Ref No/IPH/21/07/2023. Due to the fact that this study analyzed secondary data from patient charts, we were granted an informed consent waiver. To maintain confidentiality, the data collection tool did not include names or other personally identifiable information such as unique identification numbers.

## Consent

The authors have nothing to report.

## Conflicts of Interest

The authors declare no conflicts of interest.

## Data Availability

The data that support the findings of this study are available on request from the corresponding author. The data are not publicly available due to privacy or ethical restrictions. Data are available upon reasonable request. Access to data upon which the results are based can be provided upon reasonable request to the corresponding author.
